# Evaluation of artemether-lumefantrine efficacy in the treatment of uncomplicated malaria and its association with *pfmdr1*, *pfatpase6* and *K13*-propeller polymorphisms in Luanda, Angola

**DOI:** 10.1186/s12936-015-1018-3

**Published:** 2015-12-16

**Authors:** Kinanga Kiaco, Joana Teixeira, Marta Machado, Virgílio do Rosário, Dinora Lopes

**Affiliations:** Unidade de Parasitologia Médica, Instituto de Higiene e Medicina Tropical, Universidade Nova de Lisboa, Rua da Junqueira 100, 1349-008 Lisbon, Portugal; Global Health and Tropical Medicine (GHTM), Instituto de Higiene e Medicina Tropical, Universidade Nova de Lisboa, Rua da Junqueira 100, 1349-008 Lisbon, Portugal; Serviços de Saúde das Forças Armadas Angolanas, Estado Maior General das Forças Armadas, Luanda, Angola

**Keywords:** *Plasmodium falciparum*, Artemether-lumefantrine, Therapeutic efficacy, Polymorphisms, *pfmdr1*, *pfatp6*, *pfK13*, Angola

## Abstract

**Background:**

Drug resistance in *Plasmodiumfalciparum* has posed an obstacle to effective treatment and challenges many malaria control programmes in endemic areas. In Angola, until 2003, chloroquine (CQ) was used as first-line therapy for uncomplicated malaria. It was replaced initially by amodiaquine and, in 2006, by artemisinin-based combination therapy (ACT) with artemether-lumefantrine (AL, Coartem^®^). Efficacy study of ACT, conducted in Angola between 2004 and 2005, showed a baseline efficacy of ≈99 %.

**Methods:**

103 malaria patients were enrolled according to WHO proceedings. Patients were followed up with clinical and parasitological evaluations for 28 days, parasite density and identification was evaluated by microscopy, the *pfmsp2* were genotyped by nested-PCR, to distinguish parasite recrudescence from new infections; the polymorphisms at codons 86 and 1246 of *pfmdr1* gene, and 769 of *pfatp6* gene were assessed by PCR–RFLP and sequencing for *pfk13*-propeller genotype.

**Results:**

The cure rate was 91.3 %. The obtained results showed that from 103 patients, 12.6 % (n = 13) still had parasitaemia 1 day after the treatment was finished. On day 0, of the 94 evaluated samples, wild-type alleles were identified in 73.4 % (n = 69) for *pfmdr1* N86Y position and only one sample carried the mutant allele (Y) for *pfmdr1* 1246; 14 % of these samples showed increased *pfmdr1* copy number; 100 % (n = 21) had wild-type allele of *k13* gene in all the studied positions.

**Discussion:**

These results showed changes in parasite profile susceptibility to AL in comparison to the baseline data from 2002 to 2004 and on the genotyping characteristics; the clinical outcome after treatment with AL did not link a particular genotype with treatment failure; observed changes do not provide sufficient evidence for a treatment policy change, but they suggest that a carefully monitoring is needed in this area.

## Background

Malaria is the leading cause of morbidity in countries where it is endemic; an estimated 3.3 billion people are at risk of being infected with malaria and developing disease worldwide [1, 2] though, at present, some malaria control programmes are being successful [3]. Malaria control programmes currently comprise integrated interventions, including vector control with use of mosquito nets and spraying with insecticides, intermittent preventive treatment in pregnancy (IPT), early diagnosis and effective treatment of clinical cases [4]. The reports of World Health Organization (WHO) refer that malaria mortality rate declined by 47 % between 2000 and 2013 globally and 54 % in the WHO African region due to malaria control interventions [2].

Malaria remains a public health challenge in Angola with changes in mortality over the last 3 years. According to an unpublished report (2014) of National Malaria Control Programme (NMCP), between 2010 and 2012 deaths attributed to malaria decreased from 8114 to 5736, but in 2013, 1,999,868 cases and 7300 deaths due to malaria were reported [2]. In this endemic country, malaria infection is still the major cause of morbidity and mortality, accounting for 25 % of health care demands, 20 % of hospitalization requirements, 40 % of perinatal death, 25 % of maternal death and is the main reason of absenteeism, both at work and in schools [5].

An evaluation of anti-malarial efficacy of chloroquine (CQ), amodiaquine (AQ) and sulfadoxine-pyrimethamine (SP) carried out by the Angolan NMCP, in the period between 2000 and 2005, revealed treatment failure of 57.7, 3.8 and 15.4 %, respectively. Studies of artemether-lumefantrine (AL) and artesunate + amodiaquine (ASAQ), efficacy, conducted in 2004 and 2005, revealed efficacy of ≈99 % [6, 7]. Based on these results and on WHO recommendations, ACT was adopted for uncomplicated malaria treatment in 2006, with AL as the first-line drug combination [8, 9].

Despite the WHO recommendation of ACT, there are worrying signs associated with loss of efficacy. Existence of isolates from different areas with markedly reduced susceptibility to ACT was reported. A loss of efficacy of ACT in western Cambodia, with slower parasitological responses to anti-malarial drugs, principally an unusually high failure rate of artesunate + mefloquine, suggesting the emergence of artemisinin resistance, were published in 2009. In addition, prolongation of parasite clearance time (PCT) has been reported on the Thai-Myanmar border, indicating that artemisinin resistance could be spreading outside Cambodia [4, 10, 11].

The genetic basis of artemisinin resistance is not yet known; several polymorphism as *Plasmodium falciparum* multidrug resistant 1 (*pfmdr1*) gene copy number, or specific point mutations in its codons *86*, *184*, *1034*, *1042* and *1246* have been associated with the susceptibility to this class of anti-malarials [4, 12]. Certain molecular markers have been linked with resistance to lumefantrine, namely the *pfmdr1* copy number and the *pfmdr186N* allele [13, 14]. Multiple copies of the *pfmdr1* gene has been linked with lumefantrine and mefloquine resistance and to the risks of treatment failure with artesunate–mefloquine and AL in Southeast Asia, while mutations at the codon 86 of the *pfmdr1* gene can modulate lumefantrine efficacy [14, 15]. Polymorphisms in the gene *pfatp6* can also mediate changes in artemisinin susceptibility; specially the mutation S769N [13, 16, 17]. According to the WHO, a molecular marker of artemisinin resistance was recently identified, in studies in Cambodia where specific mutations in the Kelch 13 (*k13*) propeller domain were found to be associated with delayed parasite clearance; this may open new possibilities for tracking resistance to artemisinins [18–20].

The present study aims to evaluate the therapeutic efficacy of Coartem^®^ for the treatment of uncomplicated *P.falciparum* infection, in Luanda, Angola, and to analyse the polymorphisms in *pfmdr1, pfatp6,* and *K13* genes previously associated to susceptibility to AL, 6 years after its introduction on this area.

## Methods

This was an interventional prospective single cohort study conducted in Luanda, Angola, considered as malaria mesoendemic stable area. Blood sample collection was carried out in 2011, 2012 and 2013 in the period of high malaria transmission (between April and June). Patients were recruited and followed at three health centres: a Catholic Church health care unit (Hospital Divina Providência) and two military health units (Clínica do Exército and Instituto Superior Técnico Militar). Subjects were selected among children and adults with confirmed malaria infection, based on the WHO 2009 guidelines for drug efficacy study [21].

Patients were eligible to participate, if older than 6 months of age, axillary temperature of at least 37.5 °C or history of fever within the last 24 h, with symptoms compatible with malaria, and infection with *P*. *falciparum* confirmed by microscopy. Further, other criteria were included: (a) to have non-complicated malaria (according to a medical evaluation and according to asexual parasitaemia); (b) to have an initial parasite density between 1000 and 100,000 asexual parasites/μL; (c) exclusion of the concomitant diseases, after a clinical interview and examination; (d) voluntary participation in the study with written informed consent; (e) commitment to attend the follow-up examination on indicated days.

Patients with evidence of severe malaria or any other disease, presence of severe malnutrition or with appearance or history of adverse effects due to any anti-malarial drug, to other medicines, or to other causes were excluded from the study. Patients who did not meet these basic enrolment criteria were treated in accordance with routine practice of each institution. Patient’s selection was conducted by a skilled team, including physicians, nurses, microscopy’s technicians and one epidemiologist.

Ethical approval was obtained from the Ethic Committee of National Public Health Institute (Angolan Ministry of Health). All participants received information about study objectives and protocols and informed written consent was obtained from all eligible participants/parents or guardians.

Sample size should be calculated based on the expected proportion of treatment failures in the studied population [21]. As this information was not available for this area, a clinical failure rate of 50 % (P = 0.5) was assumed. A confidence level of 95 % and precision of 10 % were considered. In this study, a minimum of 96 included patients are required [21].

### Clinical analyses

Patients were treated with six doses of AL (20 mg artemether and 120 mg lumefantrine tablets; Coartem^®^, Novartis, Basel Switzerland). For adults, four tablets were administered twice a day; doses were adjusted according to weight for children under 35 kg [8]. At Hospital Divina Providência, children up to 12 years old stayed for direct observation (DOT) at the clinic, for the 3 days of medication. For patients from the remaining clinics, Coartem^®^ was given to the patient to be taken at home. Patients were recommended to take the tablets with fatty food or milk to improve their absorption [8].

Clinical assessment was performed on recruitment (day 0) and on each follow-up day (days 1, 2, 3, 7, 14, 21 and 28). Thick blood films stained with Giemsa were prepared and examined [22], on each day and capillary blood sample was collected on filter paper for DNA analysis [23, 24]. The molecular analysis was performed at Instituto de Higiene e Medicina Tropical (IHMT), in Portugal.

Outcomes were classified according to WHO anti-malarials drug efficacy test guidelines 2009 [21] as: ‘Early Treatment Failure’ (ETF) in which there are danger signs or failure to reduce parasitaemia levels by day 3; Late Treatment Failure (LTF), which consists of two subcategories: ‘Late Clinical Failure’ (LCF) in which there are danger signs or parasitaemia and fever occurring between days 4 and 28, and ‘Late Parasitological Failure’ (LPF) in which there is parasitaemia without fever between days 4 and 28; ‘Adequate clinical and parasitological response’ (ACPR) is the absence of parasitaemia on day 28, irrespective of axillary temperature, in patients who did not previously meet any criteria of treatment failure [21]. Patients meeting criteria of treatment failure ended the study and were treated with intravenous quinine. Signs, symptoms and any adverse events that developed after drug intake were observed and reported during clinical examination at the follow up days.

### Molecular analysis

Extraction of DNA from dried blood spot on filter paper was carried out using phenol–chloroform method previously tested [25, 26]. *Plasmodium falciparum* nested-PCR was performed according to the protocol described by Snounou et al. [27, 28], for identification of *Plasmodium* species and for merozoite surface protein2 (*pfmsp2*—FC and IC families*)* genotyping of on D0 and to positive samples from post-treatment (after D7) in order to distinguish parasite recrudescence from reinfection [29, 30]. Recrudescence was defined as the presence of at least one matching parasite population from day 0, while reinfections were defined as the absence of any matching parasite population from D0.

### *pfmdr1* and *pfatp6* genotyping

The analysis of polymorphisms of the parasite, based on mutations at positions 86 and 1246 of *pfmdr1*, and 749 of *pfatp6* genes were performed by conventional RFLP-PCR [31]. ApoI (New England Biolabs) was used to identify *pfmdr1* 86N, EcoRV (New England Biolabs) assessed to the 1246Y alleles and enzyme RsaI (New England Biolabs) for *pfatp6* 769S allele [31, 32]. Restriction fragments were analysed by electrophoresis using 2 % agarose (SeaKem^®^ LEAgarose, ref. 50004) gel stained with ethidium bromide (Sigma-Aldrich E1510) and visualized under UV [27, 33].

The variation of *pfmdr1* gene copy number was evaluated using real-time PCR (iCycle IQ™ (BIO-RAD), as previously described [15, 33, 34]. Briefly, real-time PCR amplification was performed under the following conditions: 10 min of pre-incubation at 95 °C followed by 40 cycles for 15 s at 95 °C and 1 min at 60 °C. PCR reactions were carried out in 20 µL final volume in a 96-well plate (Bioplastics, ref. B50501), with 7.5 µL of iQ™ SYBR^®^ Green Supermix (BIO-RAD, ref. 170-8880), 300 nM primers and 2 µL de DNA was added to the mixture. All samples were run in triplicate and the DNA of *P*. *falciparum* strains, 3D7 and Dd2 were used as single and multiple copy calibrators, respectively. *pfmdr1* copy number was calculated using a comparative threshold method (2−^ΔΔCt^ method) [34]. For this study, it was considered increased *pfmdr1* copy number when the sample had *pfmdr1* folds >1.5 by RT-PCR.

### Sequencing the *K13*-*propeller* domain

The *K13*-propeller domain was amplified according to previously described methods [20], for identification of the polymorphisms of codons G449A, N458Y, T474I, A481V, Y493H, T508 N, G533S, N537I, R539T, I543T, P553L, R561H, V568G, P574L, C580Y and D584V. Double-strand sequencing of PCR products was performed by Stabvida Genomics LAB in Lisbon. Sequences were analysed with Multalin software to identify specific SNP combinations [19, 20].

### Statistical analysis

Data were entered and analysed using Epi-info 7 version. Mixed infections thus contribute to the prevalence of both alleles were excluded of all statistical analyses.

## Results and discussion

### Sample selection

During the 3 years of study, a total of 3628 patients (adult and children) were screened for malaria parasites in the selected health care units. Of these, 972 (26.8 %) had positive microscopic malaria diagnosis; this result shows the need to consider the diverse causes of febrile syndrome in malaria endemic regions. Initially, 122 patients were selected and from these, nineteen were excluded: two vomited persistently, nine were lost during the follow-up period and 8 had a negative PCR at day 0. A total of 103 patients met the required criteria and were enrolled in the study; and from these 21 (20.4 %) were children under 5 years of age.

Treatment was well tolerated, no patients presented relevant complaints or treatment adverse effects.

### Patients follow-up

In this the study, in 2011, 37 patients were enrolled, 33 in 2012 and 33 in 2013, giving a total sample of 103 patients, aged between 6 months to 56 years of age; 716 blood samples were collected during the follow up days for molecular analysis. The group of children under 5 years of age represented 16 % of sampling (114 samples from 21 children). Distribution of observed patients and microscopy evaluation by days of follow-up is shown on Table 1.

**Table 1:** Distributions of the results by follow-up days (n = 103)

Days of follow-up	D0	D1	D2	D3	D7	D14	D21	D28
2011								
Patients observed (n)	37	37	37	36	36	35	35	35
Microscopy (+)	37 (100)	25 (67.5)	8 (21.6)	4 (11)	1 (2.8)	0	0	1 (2.8)
PCR (+)	37	34 (92)	20 (54)	13 (36)	8 (22)	4 (11)	1 (2.8)	1 (2.8)
2012								
Patients observed (n)	33 (100)	33	33	33	33	32	31	31
Microscopy (+)	33	27 (82)	6 (18)	4 (12)	2 (6)	1 (3)	0	0
PCR (+)	33	30 (91)	19 (58)	14 (42)	10 (30)	1 (3)	0	0
2013								
Patients observed (n)	33	33	33	33	33	29	29	29
Microscopy (+)	33 (100)	30 (91)	6 (18)	5 (15)	3 (9)	0	0	1 (3.4)
PCR (+)	33	33 (100)	30 (91)	21 (63.6)	17 (51.5)	11 (38)	5 (17)	3 (10)
Total								
Patients observed (n)	103	103	103	102	102	96	95	95
Microscopy (+)	103 (100)	82 (80)	20 (19)	13 (13)	6 (6)	1 (1)	0	2 (2)
PCR (+)	103	97 (94)	69 (67)	48 (47)	35 (34)	16 (17)	6 (6)	4 (4)

PCR, polymerase chain reaction; n, number of patients; (), relative frequency; (+), positive result; D, day of follow-up

### Identification of *Plasmodium falciparum* species

On D3 (one day after finishing the Coartem^®^ treatment), 13 of the 103 patients (12.6 %) had parasites detectable by microscopy, six of those patients (5.8 % of the total) still had parasites detectable by microscopy on D7. Using the PCR method, 48 patients (46.6 %) on D3 and 35 (34 %) on D7 gave positive results for presence of *P. falciparum*. Samples with positive PCR had correspondence with follow-up days with positive microscopic results for asexual forms and/or gametocytes. The high sensitivity of the PCR method to detect the parasites at sub-microscopic densities, gametocytes presence and errors in microscopic diagnostic are factors that can generally explain the difference on results obtained by microscopic and PCR methods [21, 22]. To minimize the microscopy error in this study, skilled technicians were involved. All individuals that treatment responses were identified as treatment failures were both microscopic and PCR positive at respective days.

According to the years of inclusion, it was observed that the proportion of samples with parasites detectable by PCR at day 3 and 7 increased progressively from 2011 to 2013 as showed on Table 1.

### Gametocyte carriage

Gametocytes were detected in 15.5 % (n = 16) of the patients on D0. Seven patients (6.8 %) still had gametocytes detectable on D7 and were still found in three patients on D14, in one on D21 and one on D28, after the asexual parasites had been cleared. The presence of gametocytes on D0 can suggest the usual existence in the endemic areas of long-lasting infections, initially asymptomatic with low parasitaemia, which can turn manifest when there is debility of host defense or new additional infection. This condition promotes maintenance of disease transmission in the community [35].

### Fever clearance

The average fever clearance time was ≈24 h after the initial dose of treatment, 97 % of the patients had no fever at the second day and 99 % were not febrile on the third day of medication (D2). One patient was still febrile and with parasitaemia on D2, being classified as ETF. By D3 the totality of patients was cleared of fever. During the follow-up period 13 and 6 patients still had parasite positive blood smear on days 3 and 7 respectively, not presenting any clinical signs.

### Treatment outcomes

Of the 103 patients, in the study, 90.3 % (n = 93) were classified as ACPR. Nine patients exhibited parasitaemia after treatment with AL. The global failure rate before PCR correction was 9.7 % (n = 10). One patient, under 5 years of age, had persistent fever and parasitaemia until day 2, which was classified as ETF, according to WHO protocol, eight patients were classified as LPF, being six asymptomatic patients with parasite positive blood smear on D7 and two asymptomatic with blood parasitaemia on D14 and D28 respectively. One febrile patient presented parasites on D28 and was classified as LCF. After PCR correction, the overall cure rate was 91.2 % (n = 94); recrudescence was found in eight patients and reinfection in one. Among the 21 children ≤5 years of age included in the study, five (23.8 %) presented treatment failure and three of these had anti-malarial medication under direct observed regime, at Divina Providência Hospital.

According to the age group, the frequency of ACPR was 76.2 % in patients ≤5 (n = 16/21) years and 95.1 % (n = 78/82) in those aged >5 years; there was no statistically significant association between treatment outcomes and age groups (p = 0.334). The treatment outcome is shown in Table 2.

**Table 2:** Treatment outcomes for patients treated with Artemether-Lumefantrine

Clinical outcome	Total	Age group	
Initially selected	122	28 ≤ 5 years	94 > 5 years
Number of patients: n			
PCR uncorrected responses			
Withdrawn + Lost, n (%)	19 (15.6)	7 (25)	12 (12.8)
Eligible; n (%)	103 (100)	21 (20.4)	82 (79.6)
Global failure; n (%)	10 (9.7)	5 (23.8)	5 (6.1)
ETF; n (%)	1 (1 %)	1 (4.8)	0
LCF; n (%)	1 (1)	0	1 (1.2)
LPF; n (%)	8 (7.8)	4 (19)	4 (4.9)
ACPR; n (%)	93 (90.3)	16 (76.2)	77 (94)
PCR corrected responses			
Eligible; n (%)	103 (100)	21 (20.4)	82 (78.6)
Global Failure; n (%)	9 (8.7)	5 (24)	4 (5)
ACPR; n (%)	94 (91.2)	16 (76)	78 (95)
Recrudescence; n (%)	2 (1.9)	1 (4.8)	1 (1.2)
Reinfection; n (%)	1 (1)	0	1 (1.2)

*PCR* polymerase chain reaction, *n* number of patients, *ACPR* adequate clinical and parasitological response, *ETF* early treatment failure, *LTF* late treatment failure

The WHO considers the proportion of patients with parasitaemia on day 3 of treatment as routine indicator in monitoring of the *P. falciparum* sensitivity to artemisinin and its derivatives, defining suspected resistance to artemisinin as an increase in parasite clearance time, as evidenced by 10 % or more cases with parasites detectable on day 3 of treatment with ACT. Changing of anti-malarial treatment policy is recommended when the treatment failure rate exceeds 10 % [4, 10, 11].

In this study, 12.6 % (n = 13) of the 103 followed patients had still microscopic parasites detectable on D3 and failure rate of 8.8 % was met. Considering WHO parameters, the results met in this study might suggest an elongated parasite clearance time [4, 11]. The failure rate of 8.8 % is considered higher in comparison to the failure of 1.2 % found in 2004 as baseline for AL efficacy in Angola [6, 7] and to the average failure rates of <5 % are often reported in the majority of studies conducted in Sub-Saharan African region [2–4, 36]. In the neighbouring countries, average failure rates reported were: 2.8 % from Congo, 2.4, 6.7 and 2.9 % from Democratic Republic of Congo, Zambia and Tanzania, respectively [2, 3, 37]. Failure rates >10 % were also reported as maximal failure rates among studies carried out in African countries such as Burkina Faso (12.5 %) in 2007, Ghana (13.8 %) in 2006 and Malawi (19.5 %) [2, 38] and a rate of 11.6 % was reported by Plucinski et al. in the Zaire province in Angola in 2013 [39]. The adjusted—PCR cure rate of 91.2 % met in this study is low comparing to the finding of the pooled analysis of data from 31 studies conducted by Venkatesan et al, which reported AL overall median ACPR of 94.8 % and regional medians of 93.8 % for East Africa, 96.2 % for West Africa and 95.2 % for Asia/Oceania [40, 41]. Considering that the majority of the referred studies were completed before 2010, more studies are needed to update the understanding of the current efficacy of AL in Africa. The cure rate (91.2 %) found in this study suggests that, in accordance with WHO parameters, AL is still effective as first-line drug for uncomplicated malaria treatment in Angola, but an regular monitoring is necessary.

### Molecular analysis

#### Merozoite surface protein2 (*pfmsp2)* genotyping

*pfmsp2* genotyping was performed for all samples on day 0 and for those with microscopic positive result from D14 to differentiate between new infection and recrudescence. The *pfmsp2* genotyping was successfully assessed in 96 samples from (D0). Of these, 32 samples (33.3 %) were collected in each year, respectively 2011, 2012 and 2013. From the *pfmsp2* genotyping analysis, 72 % (n = 69) of the 96 samples had just one of the studied alleles (FC27 or IC1), therefore termed as monoclonal infections and the IC1 family was predominant with 62.3 % (n = 43) of monoclonal samples.

The remaining mixed infections, with FC and IC families, were found in 28 % (n = 27) of the analysed samples. Polyclonal infections were found in 37.5 % of cases (n = 36/96) on day 0. A progressive elimination of mixed variants post-treatment was noticed. After day 3, no more samples with mixed families were detected. The genotyping of *pfmsp2* in the *3* microscopic positive samples from day 14 demonstrated that 2 had the matching allelic bands of day 0, indicating recrudescence. One positive sample on day 28 did not have the matching allelic bands of day 0, indicating reinfection. Seven of eight amplified samples from patients with treatment failure carried IC1 family alleles. The statistic tests did not evidence significant association between *msp2* families identified and treatment responses (P = 0.115). In this study, the reduced number of samples with parasites detectable from day 14 constitutes a limitation for relevant conclusions on recrudescent and new infection rates.

### Molecular markers for drug resistance

#### Prevalence of SNP at 86 and 1246 positions of *pfmdr1* gene

Gene fragments containing the SNP 86 (N/Y) and 1246 (D/Y) were successfully amplified on D0 in 94 and 96 samples, respectively. For the analysis of *pfmdr1* 86 SNP, out of 94 analysed samples, 28 (29.8 %) were collected in 2011, 33 (35.1 %) isolates collected in 2012 and 33 (35.1 %) in 2013. The global ranges of frequency were 73.4 % for the wild allele (N86), 18.1 % for the mutant (86Y) and 8.5 % for the mixed alleles (N + Y). The ranges of frequencies by years for *pfmdr1* N86 allele were 68 % (19/28) in 2011, 69.7 % (23/33) in 2012 and 82 % (27/33) in 2013. In the respective years, the frequencies of the mutant 86Y were 21, 18.2 and 15 %; and the mixed infections was found in 11, 12 and 3 % in 2011, 2012 and 2013, respectively.

These results show a predominance of the wild-type allele for the *pfmdr1* 86 position, there was no significant association between the presence of *pfmdr1* 86 polymorphism and the year o collection (p = 0.869). The *pfmdr1* D1246 was the predominant allele in the isolates for all time points, and one isolate only, collected in 2012, presented the mutant allele 1246Y, this isolate was obtained from a patient classified as ACPR and which carried a mixed 86 N + Y allele.

The results of this study are in agreement with those reported by Fançony et al. in northern Uíge province of Angola in 2009, where rates of 68.1, 17.4 and 14.5 % for N86, 86Y and 86 N + Y, respectively, were encountered [42]; however, differ from those met by Menegon et al., in Uíge province earlier, in 2004, where 86Y was the most prevalent (68.2 %) [43] and those from patients assisted in paediatric hospital in Luanda in 2007/2008, which reported a 86Y frequency of 61.3 % [32].

The analysis of pooled data from 31 studies (1995–2010) conducted by Venkatesan et al. in 2014 reported rates of 37 % (N86), 44 % (86Y) and 19 % (86 N + Y) for Est Africa, 51 % (N86), 14 % (86Y) and 34 % (86 N + Y) for West Africa and 71 % (N86), 29 % (86Y9) and 0 % (86Y + Y) in Asia/Oceania [41].

Variations in drug exposure seem to affect the *pfmdr1* genotype profile trend. Recently, several field studies have suggested that AL appeared to select the N86 wild-type allele of *pfmdr1* gene associated to reduced susceptibility to lumefantrine. Also, the predominance of this wild-type allele can be related to the elimination of CQ and AQ monotherapy [44, 45]. Therefore, the results met in this study could be a reflection of changes occurring in the local parasite populations, including the progressive predominance of the wild-type N86 allele, associated to the introduction of ACT in Angola since 2006, with elimination of the CQ monotherapy.

For the *pfmdr1* 1246 polymorphism, the wild-type (D1246) was present as the major allele in the isolates. Just one isolate collected in 2012 presented the mutant allele 1246Y, this isolate was from a patient classified as ACPR and which carried a mixed 86 N + Y alleles. The results of this study are in accordance with those found in a majority of studies in the African region where usually the mutant 1246Y is not frequent [41], but differ with the finding in samples from Kinshasa—DRC (2008) which reported frequencies of 15.5 % (n = 16/103) for *pfmdr1* 1286Y and 76.7 % for D1246 alleles [46, 47].

Detection of the *pfmdr1* 1246Y in isolates from West Africa (Guinea Bissau), in DRC (central Africa) an in this study (Angola) reveals a emergence of this genotype previously considered rare in Africa but common in South America. The presence of these genotypes on this area may due to the increased migration phenomenon and globalization.

### Prevalence of polymorphisms at position 769 of *pfatp6* gene

SNP at 769 (S769N) position of the *pfatp6* gene were assessed in all samples (103), none of the isolates displaying the 769 N mutation; it should be noted that even the recrudescent samples exhibited the wild-type S769 haplotype. This result coincides with Menegon et al., where wild-type allele S769 was found in all isolates collected in 2004. Similar results were reported from studies conducted in the Africa Region [13, 15].

### Prevalence of polymorphisms on pfK13-propeller gene

Regarding *pfK13*-*propeller* gene, all samples (n = 21) processed by sequencing exhibited the wild type allele in all analysed positions, including in SNPs considered associated to artemisinin resistance (Y493H, R539T, I543T and C580Y). The results of this study are in agreement with those reported by Plucinski and by Escobar et al., where wild-type alleles were found at the rendered positions of *pfk13* gene, in isolates from Angola and Mozambique [39, 48]. The frequencies of genetic markers associated with drug resistance are shown on Table 3.

**Table 3:** Prevalence of genetic markers associated with drug resistance

Year	Gene	SNP’s	n	Allele %		
				Wild-type	Mutant	Mixed allele
2011	*pfmdr1*	N86Y	28	68 % (n = 19)	21 % (n = 6)	11 % (n = 3)
		D1246Y	31	100 %	0	0
		>copy	4/32 (12.5 %)			
	*pfatp6*	S769N	37	100 %	0	0
	*K13*	Y493H	5	100 %	0	0
		R539T	5	100 %	0	0
		I543T	5	100 %	0	0
		C580Y	5	100 %	0	0
2012	*pfmdr1*	N86Y	33	69.7 % (n = 23)	18.2 % (n = 6)	12.1 % (n = 4)
		D1246Y	32	97 % (n = 3)	3 % (n = 1)	0
		>copy	4/29 (13.8 %)			
	*pfatp6*	S769 N	33	100 %	0	0
	*K13*	Y493H	7	100 %	0	0
		R539T	7	100 %	0	0
		I543T	7	100 %	0	0
		C580Y	7	100 %	0	0
2013	*pfmdr1*	N86Y	33	82 % (n = 27)	15 % (n = 5)	3 % (n = 1)
		D1246Y	33	100 %	0	0
		>copy	5/32 (15.6 %)			
	*pfatp6*	S769 N	33	100 %	0	0
	*K13*	Y493H	9	100 %	0	0
		R539T	9	100 %	0	0
		I543T	9	100 %	0	0
		C580Y	9	100 %	0	0
Total	*pfmdr1*	N86Y	94	73.4 (n = 69)	18.1 % (n = 17)	8.5 % (n = 8)
		D1246Y	96	99 (n = 86)	1 (n = 1)	0
		>copy	13/93 (14 %)			
	*pfatp6*	S769N	103	100 % (n = 103)	0	0
	*K13*	Y493H	21	100 %	0	0
		R539T	21	100 %	0	0
		I543T	21	100 %	0	0
		C580Y	21	100 %	0	0

*pfmdr1*, *Plasmodium* *falciparum* multidrug resistance 1 gene, SNP, Single nucleotide polymorphism, *Pfatp6*, *Plasmodium* *falciparum* sarco/endoplasmic reticulum calcium-ATPase6 gene, *K13*, mutations in the PF3D7_1343700 kelch propeller domain (‘K13-propeller’); wt, wild type allele, mixed, mix infections, >copy, *pfmdr1*amplifyed (with >1 copy)

### *pfmdr1* gene copy number

The Table 4 shows the correlation between frequencies of the analysed SNPs and treatment outcomes. Ninety-three samples were successfully analysed by real-time PCR for the detection of *pfmdr1* copy number on D0. The results demonstrated that 13 isolates (14 %) showed increased copy number, ranging from 2 to 3 copies. The majority of analysed isolates had a single copy of the gene *pfmdr1* (86 %, n = 80), 12 isolates (13 %) had two copies (1.6 ≤ 2.5 folds) and one sample (1 %) had three copies (3.44 folds). The proportions of samples with amplified *pfmdr1* by year of collection were: 12.5 % (n = 4/32) in 2011, 13.8 % (n = 4/29) in 2012 and 15.6 (n = 5/32) in 2013.

**Table 4:** Correlation between SNP frequencies and treatment outcomes

Treatment outcome	*SNPs*									*pfmdr1* >*copy*
	*pfmdr1* 86			*pfmdr1* 1246		*Pfatp6 769*		*pfK13*		
	N86	86Y	86 N + Y	D1246	1246Y	S769	769 N	wt	mutant	
ACPR	74.1 % (n = 63)	17.6 % (n = 15)	8.2 % (n = 7)	99 % (n = 88)	1 % (n = 1)	100 % (n = 93)	0	100 % (n = 12)	0	13 % (n = 12/92)
Treatment failure	66.6 % (n = 6)	22.2 % (n = 2)	11.1 % (n = 1)	100 (n = 7)	0	100 % (n = 10)	0	100 % (n = 9)	0	11 % (n = 1/9)
Total (n)	69	17	8	95	1	103	0	21	0	13

*pfmdr1*, *Plasmodium* *falciparum* multidrug resistance 1 gene, SNP, Single nucleotide polymorphism, *Pfatp6*, *Plasmodium* *falciparum* sarco/endoplasmic reticulum calcium-ATPase6 gene, K13, mutations in the PF3D7_1343700 kelch propeller domain (‘K13-propeller’); wt, wild type allele; mixed, mix infections, >copy, *pfmdr1* amplifyed (with >1 copy)

According to AL treatment outcomes, of the 13 samples with *pfmdr1* increased copy number, 92.3 % (n = 12/13) were from patients classified as ACPR. The nine patients classified as treatment failure exhibited a single copy of *pfmdr1* whenever tested, whilst the patient initially classified as LCF was finally confirmed as a new infection had presented two copies of the gene on D0 and a single copy for D28.

An increase in the copy number of *pfmdr1* gene has been associated with clinical failures and within vitro resistance to aryl-amino-alcohols, particularly mefloquine (MQ), quinine (QN), and halofantrine, but also to lumefantrine [15, 47, 49]; the results of this study didn’t show a statistically significant association between increase in copy number of the gene *pfmdr1* and therapeutic response to Coartem^®^. However, the frequency of isolates with increase copy number of *pfmdr1* gene found in this study seems higher in comparison to average reported in various studies from African region [41].

According to other studies the *pfmdr1* copy amplification has been rarely found in the African region [41, 50]. There is no data published about *pfmdr1* copy number from Angolan patients; however from other countries, and from a total of 131 isolates collected in 2005–2009 from West Africa (n = 99) and Central Africa (n = 32), only 4 had *pfmdr1* copy number amplification. Menard et al. in Cameron in 2009 did not identify samples with *pfmdr1* gene amplification, and similar results were reported by Vaughan-Williams et al. in Kwazulo Natal, South Africa, in 2012 [50, 51].

## Conclusions

Relatively to Coartem^®^ treatment response, the failure rate of 8.8 % is higher in comparison to that of 1.2 % found in the 2004 study for AL efficacy in Angola [2, 39], and the median failure rates of <5 % often reported in the majority of studies conducted in sub-Saharan African region; however, the cure rate (91.2 %) of this study suggests that AL is still effective as first line drug for uncomplicated malaria treatment in Angola, in accordance to the WHO parameters [4, 21].

The predominance of the wild-type allele for the *pfmdr1* 86 and 1246 positions found in this study is in agreement with those reported in various studies conducted in the African region [41]. Recently, several field studies have suggested that AL eliminates the mutated parasite population allowing the wild-type to become predominant. Considering that ACT was introduced in Angola from 2006, and after 7 years of AL regime implementation, the results met in this study could reflect changes occurring in the local parasite populations, including progressive elimination of mutant *86Y* allele previously selected by CQ. Detection of the *pfmdr1* 1246Y in this study as well as in others studies of isolates from West Africa (Guinea Bissau) and DRC (Central Africa) seems to indicate a emergence of this genotype previously considered rare in Africa, may be due to globalization and migration. The frequency of isolates with increase copy number of *pfmdr1* gene found in this study seems higher in comparison to others reported in various studies from the African region where copy amplification of this gene has been rarely found. The analysis of the clinical outcomes after treatment with Coartem^®^ did not link a particular genotype with treatment failure met in this study.

Globally, the result of this study suggests a change of the profile of the parasite population circulating in Angola, reflected by a decreased susceptibility to AL and a relative alteration in *pfmdr1* genotyping in comparison to many studies previously realized in the area. However, the low number of patients in the study and with treatment failure could be a limitation for a consistent conclusion.
